# Alteration of mitochondrial biogenesis promotes disease progression in multiple myeloma

**DOI:** 10.18632/oncotarget.22740

**Published:** 2017-11-27

**Authors:** Xin Zhan, Wenjie Yu, Reinaldo Franqui-Machin, Melissa L. Bates, Kalyan Nadiminti, Huojun Cao, Brad A. Amendt, Yogesh Jethava, Ivana Frech, Fenghuang Zhan, Guido Tricot

**Affiliations:** ^1^ Division of Hematology, Oncology, and Blood and Marrow Transplantation, Department of Internal Medicine, University of Iowa, Iowa City, IA, USA; ^2^ Department of Anatomy and Cell Biology University of Iowa, Iowa City, IA, USA; ^3^ Molecular & Cellular Biology Program, University of Iowa, Iowa City, IA, USA; ^4^ Department of Health and Human Physiology, University of Iowa, Iowa City, IA, USA; ^5^ Department of Endodontics University of Iowa, Iowa City, IA, USA

**Keywords:** multiple myeloma, mitochondria biogenesis, iron, disease progression

## Abstract

Many cancers, including multiple myeloma (MM), retain more cytosolic iron to promote tumor cell growth and drug resistance. Higher cytosolic iron promotes oxidative damage due to its interaction with reactive oxygen species generated by mitochondria. The variation of mitochondrial biogenesis in different stages of MM disease was evaluated using gene expression profiles in a large clinical dataset. Sixteen of 18mitochondrial biogenesis related gene sets, including mitochondrial biogenesis signature and oxidative phosphorylation, were increased in myeloma cells compared with normal plasma cells and high expression was associated with an inferior patient outcome. Relapsed and drug resistant myeloma samples had higher expression of mitochondrial biogenesis signatures than newly diagnosed patient samples. The expression of mitochondrial biogenesis genes was regulated by the cellular iron content, which showed a synergistic effect in patient outcome in MM. Pharmacological ascorbic acid induced myeloma cell death by inhibition of mitochondria oxidative phosphorylation in an *in vivo* model. Here, we identify that dysregulated mitochondrial biogenesis and iron homeostasis play a major role in myeloma progression and patient outcome and that pharmacological ascorbic acid, through cellular iron content and mitochondrial oxidative species, should be considered as a novel treatment in myeloma including drug-resistant and relapsed patients.

## INTRODUCTION

Multiple myeloma (MM) is a difficult to cure plasma cell tumor. It is the second most common blood malignancy. High-dose chemotherapy approaches, including proteasome inhibitors, immunomodulatory drugs (IMiDs), alkylating agents, and glucocorticoids with tandem auto-transplants in recently diagnosed MM patients have led to complete remissions (CRs) in the large majority of newly diagnosed patients with MM. However, many patients who achieve CR subsequently relapse. We recently showed that pharmacological ascorbic acid (PAA) as a pro-oxidant agent selectively kills MM cells by generating ascorbyl- and H_2_O_2_ radicals in cancer cells [1]. PAA-induced cell death depends on cellular iron content and cleavage of apoptosis-inducing factor 1 (AIF1), a mitochondrial biogenesis related gene [1]. Furthermore, increasing intracellular steady-state pro-oxidant levels in stem-like and mature MM cells using a mitochondrial-targeting agent decyl-triphenylphosphonium (10-TPP) promotes cancer cell death [2]. Though many pre-clinical studies for targeting mitochondria have been reported in MM [3, 4], it remains unclear whether mitochondrial biogenesis is beneficial or detrimental for tumor cells. A better understanding of the genetic makeup of mitochondria in MM cells is required if we want to use mitochondria as specific targets.

In cancer cells, mitochondria generate ATP through both oxidative phosphorylation (OXPHOS) and aerobic glycolysis (the Warburg effect) for cell proliferation [5]. Both mitochondrial biogenesis and mitophagy (mitochondrial autophagy) control mitochondrial content and metabolism [6, 7]. In cancer cells, mitochondrial biogenesis is generally positively regulated by oncogenes and/or negatively by tumor suppressor genes, such as c-Myc and p53 respectively [8, 9]. Mitochondria are organelles where heme and Fe-S clusters are synthesized [10]. Mitochondria are also the major source of reactive oxygen species (ROS), which may induce oxidative damage to DNA, lipids and proteins [8]. Iron acts as an oxidant or reductant by accepting or donating single electrons in a variety of biochemical reactions. Many cancers, including MM, have altered iron metabolism resulting in increased intracellular iron content that facilitates tumor cell growth and drug resistance. Specifically, Ferroportin 1 (FPN1), the only iron exporter in mammalian cells, is decreased in MM tumor cells leading to an increase in the labile iron pool [11]. However, the correlation between these two important pathways that are independently linked to cancer pathogenesis: mitochondrial biogenesis and iron metabolism, has not been investigated in MM disease.

As we described above, the intracellular iron content and mitochondrial biogenesis are important parameters to monitor disease progression and to design novel targeted therapy [12]. Using gene expression profiles (GEP) in a large clinical dataset, we generated seven molecular genetic subgroups and a 70-gene risk model [13, 14]. These models can predict the genetic changes and prognosis in primary MM patients. Here, we evaluate correlate variations in mitochondrial biogenesis to sensitivity to chemotherapy. We also explore whether dysregulated iron metabolism influences mitochondrial metabolism in MM disease progression. Our data support that mitochondrial biogenesis is increased in drug resistant and relapsed MM cells and PAA can overcome drug resistance via inhibition of mitochondrial oxidative phosphorylation.

## RESULTS

### Mitochondrial biogenesis signature is increased in newly diagnosed myeloma patients and associated with poor outcome

**Figure 1:**
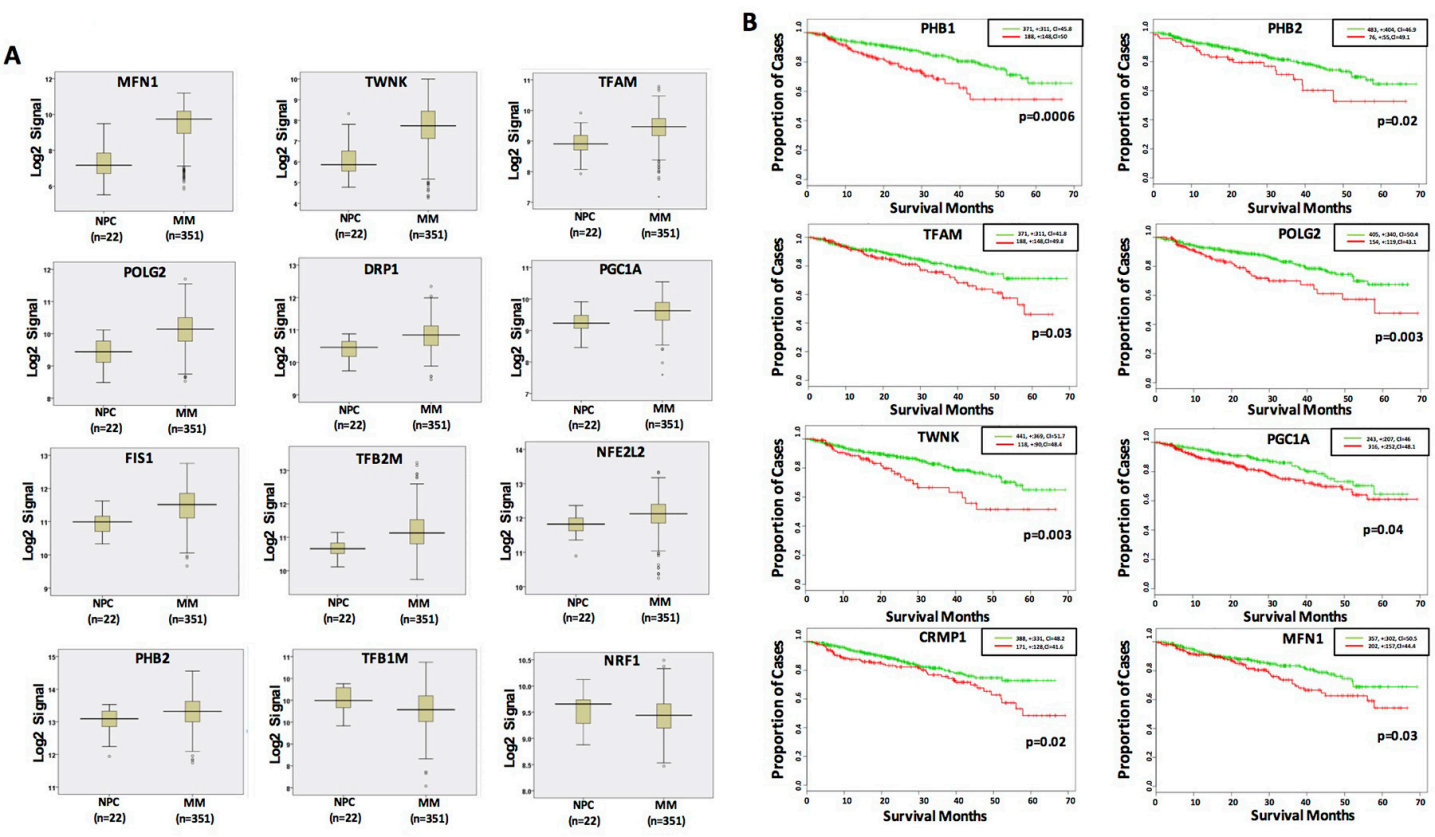
Dysregulated MitoBio genes in MM are associated with lower survival **(A)** Gene expression profiles of 12 dysregulated MitoBio genes from 22 normal plasma cells (NPC) and 351 newly diagnosed MM samples in the total therapy 2 (TT2) clinical trial. **(B)** Kaplan–Meier survival analyses of 18 MitoBio genes were performed in 559 newly diagnosed MM samples. The correlation between GEP and survival was determined by the p value and hazard ratio (HR) at the best expression signal cut-off using SurvExpress website.

Mitochondrial biogenesis (MitoBio) contains 18 genes from the following categories [12]: 1) mitochondrial membrane integrity (*PHB1* and *PHB2*); 2) mitochondrial transcription factors (*TFAM*, *TFB1M*, and *TFB2M*); 3) mtDNA replication factors [*POLGA*, *POLGB*, and *Twinkle (C10orf2)*]; 4) co-activators of MitoBio [*PPARGC1A (PGC1A)*, *PPARGC1B (PGC1B)*, and *PPRC1*]; 5) nuclear respiratory factors (*NRF1*, and *NFE2L2*), 6) mitochondrial fission mediators [*DRP1* (*DNM1L*) and *FIS1*], 7) mitochondrial fusion mediators (*MFN1* and *MFN2*); and 9) other regulators of MitoBio (*ESRRA*). We collected plasma cells from healthy donors, tumor cells from Monoclonal Gammopathy of Undetermined Significance (MGUS) and newly diagnosed MM patients using CD138 antibody magnetic beads as previously described, and performed microarrays on Affymetrix U133Plus2 platform [15]. By comparing gene expression profiles of these MitoBio genes from 22 normal plasma cells (NPC) samples and 351 newly diagnosed MM samples in the Total Therapy 2 (TT2) clinical trial, 12 of the above described 18 genes are significantly dysregulated. *MFN2*, *Twinkle*, *POLGA*, *TFB2M*, *FIS1*, *TFAM*, *PPRC1*, *PHB2*, *NFE2L2*, and *POLG2* (0.012 > *p* > 1 x 10^-10^ (ordered from the largest to smallest *p* values) are upregulated and *TFB1M* (p = 0.014) and *NRF1* (p = 0.049) are downregulated (Figure 1A).

To determine whether the elements of the MitoBio profile are linked to patient outcome, Kaplan–Meier survival analysis was performed on the Total Therapy 2 (TT2) and TT3 cohorts, which included gene expression profiles (GEP) from 559 newly diagnosed myeloma patients. The correlation between GEP and survival was determined by the p value and hazard ratio (HR) at the best expression signal cut-off using the SurvExpress website. High expression of *PHB1*, *PHB2*, *TFAM*, *POLG2*, *TWNK*, *PGC1A*, *CRMP1*, and *MFN1* genes were associated with inferior survivals in unadjusted log rank tests (Figure 1B).

**Figure 2:**
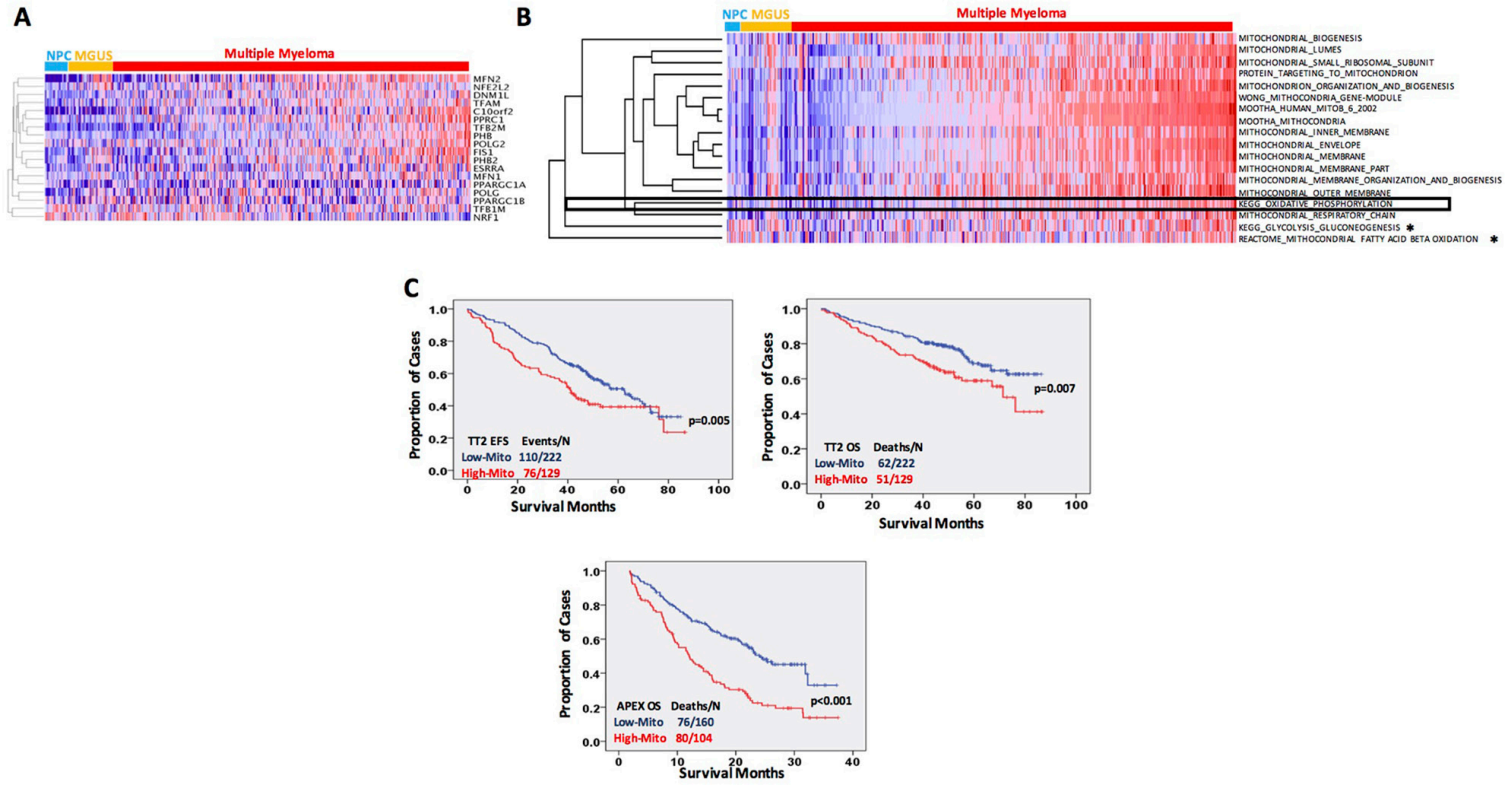
MitoBio signature is increased in newly diagnosed myeloma and links to a poor outcome **(A)** Gene expression clustergram of 18 MitoBio genes in plasma cells from 22 healthy subjects (NPC), 44 MGUS and 351 newly diagnosed MM patients. **(B)** Single–sample gene set enrichment analysis performed in 22 NPC, 44 MGUS and 351 MM GEPs for MitoBio signature clustered with other 17 mitochondrial gene sets. MM samples were pre-ordered based on the ssGSEA scores of the gene set with all genes involved in the eighteen mitochondria-related pathways (Total-ssGSEA scores). Sixteen of the 18 sets are significantly upregulated in multiple myeloma [p<0.05; ^*^ No statistic significance (p>0.05)]. **(C)** Kaplan–Meier analyses of event free survival (EFS) in TT2 cohort and overall survival (OS) in TT2 and APEX cohorts.

A supervised hierarchical cluster was performed on 22 NPC, 44 MGUS and 351 MM samples using the 18 MitoBio genes. As shown in the Figure 2A, a subgroup of MM samples showed a relative high MitoBio signature. We further analyzed the correlation of MitoBio signature with additional 17 mitochondrial gene datasets using the “KEGG Glycolysis and Gluconeogenesis” and “KEGG Oxidative Phosphorylation” as mitochondrial functional controls. A single–sample gene set enrichment analysis (ssGSEA) was performed to calculate the enrichment score for each gene dataset in the 22 NPC, 44 MGUS and 351 MM GEPs. As shown in the Figure 2B, the MitoBio signature was clustered with other 17 mitochondrial gene datasets. Sixteen of 18 mitochondrial gene datasets were significantly upregulated in MM cells compared to plasma cells derived from NPC and MGUS, only Glycolysis Gluconeogenesis and Mitochondrial Fatty Acid Beta Oxidation did not show difference between these groups (*in Figure 2B, p > 0.05). Interestingly, the oxidative phosphorylation pathway (OXPHOS pathway) was highly correlated with the MitoBio signature (p<0.0001); whereas the glycolysis gluconeogenesis was not, suggesting that MitoBio may contribute to OXPHOS pathway, but not to glycolysis, for generation of ATP in MM cells.

**Figure 3:**
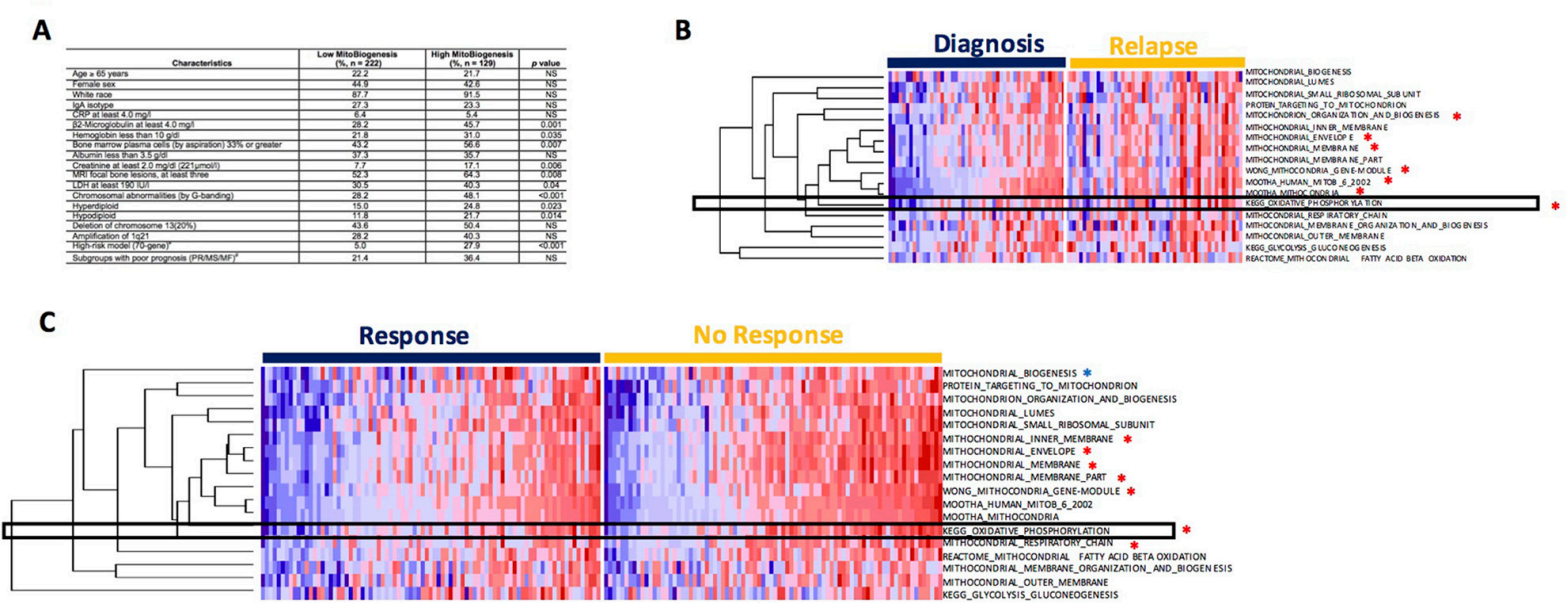
Drug resistant myeloma patients show higher expression of MitoBio gene sets **(A)** Univeriate analysis was used to analyze the correlation of MitoBio score and clinical characteristics in TT2. **(B)** Single–sample gene set enrichment analysis performed in 51 paired GEPs collected at diagnosis and at relapse from 51 MM patients enrolled in the TT2 cohort. Seven of the 18 sets show significantly upregulation (*). Samples were pre-ordered based on the Total-ssGSEA scores in diagnosis group. **(C)** Correlation of MitoBio genes with drug resistance in the APEX trial including 264 relapsed MM samples and 169 patients were treated with bortezomib. The MitoBio signature, oxidative phosphorylation and 6 other mitochondrial datasets (^*^p<0.05) are increased in bortezomib-resistant MM samples (right) compared to bortezomib-sensitive MM samples (left). Samples were pre-ordered based on the Total-ssGSEA scores in each group.

We calculated a score for each sample using the 18 mitochondrial gene datasets. A correlation between the mitochondrial-score derived from the 18 mitochondrial gene sets with patient outcome was analyzed in the 351 TT2 newly diagnosed MM samples and 264 APEX relapsed MM samples. The group with the higher mitochondrial-score had a significantly inferior event-free survival (EFS) (Figure 2C, *p* = 0.005) and overall survival (OS) (Figure 2C, *p* = 0.007) in the TT2 cohort and in the APEX cohort (Figure 2C, *p* < 0.001). The correlation between clinical parameters and MitoBio subgroups was also analyzed in the TT2 patients by a univariate analysis (Figure 3A). Patients with a high mitochondrial-score had higher levels of β2-microglobulin (p = 0.001), lower levels of hemoglobin (p=0.035), higher percentage of MM cells in bone marrow aspirate (p = 0.007), more lytic bone lesions identified by magnetic resonance imaging (MRI; p = 0.008), higher lactate dehydrogenase (LDH; p = 0.04), higher creatinine (p = 0.006), higher frequency of chromosomal metaphase cytogenetics (p < 0.001) with increased hyperdiploid (p = 0.023) and hypodiploid (p = 0.014). The high MitoBio cohort predominated in the high-risk MM group defined by the 70-gene model (p < 0.001).

### Expression of mitochondrial biogenesis genes is higher in drug resistant and relapsed patients

To investigate whether increased MitoBio signature is increased at relapse, 51 paired GEPs collected at diagnosis and again at relapse were compared using the ssGSEA analysis. The enrichment scores of 7 of 18 mitochondrial data sets are significantly increased in relapsed samples including OXPHOS pathways (Figure 3B; p < 0.01). We also investigated the correlation of 18 mitochondrial data sets with drug resistance in the APEX trial including 169 relapsed MM samples [16]. The analyses of genome-wide gene expression data showed that MitoBio and OXPHOS signatures and another 6 pathways were increased in bortezomib-resistant MM samples compared to bortezomib-sensitive MM samples (Figure 3B; p < 0.01). However, we did not find a correlation between MitoBio signature and dexamethasone sensitivity (data not shown). These results suggest that MM patients with a high MitoBio signature and an activated OXPHOS pathway are resistant to bortezomib treatment.

### Cellular iron content influences mitochondrial biogenesis and patient outcome in myeloma

**Figure 4:**
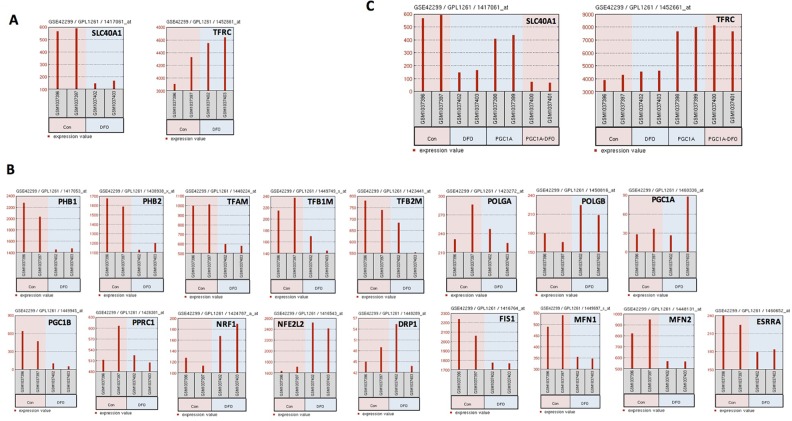
Iron influences mitochondrial biogenesis and patient outcome in myeloma **(A)** The expression *FPN1* (SLC40A, left panel) and *TFRC* (right panel) was analyzed in untreated and DFO-treated C2C12 cells determined by GEP. **(B)** *FPN1* expression (SLC40A1, left panel) and *TFRC* (right panel) was analyzed in untreated and DFO-treated C2C12 cells overexpressing PGC1A. **(C)** Gene expression of 17 MitoBio genes in untreated and DFO-treated C2C12 cells.

Disruption in iron homeostasis is often coupled with mitochondrial alteration. Because mitochondria are the major organelle to play a central role in iron-metabolism, we analyzed whether cellular iron influences MitoBio or vice versa. The expression of 18 MitoBio genes was compared in control C2C12 cells and the same cells treated with the iron chelator desferoxamine (DFO) [17]. C2C12 is an immortalized mouse myoblast cell line widely used in biomedical research. Expression of the expression of iron exporter gene *FPN1* is downregulated in C2C12 cells treated with DFO, while the iron importer gene *TFRC* is upregulated (Figure 4A). Ten of 17 MitoBio genes (the gene *Twinkle* was not found on the Chip Annotation) including *PHB1*, *PHB2*, *TFAM*, *TFB1M*, *TFB2M*, *PGC1B*, *FIS1*, *MFN1*, *MFN2*, and *ESRRA* were downregulated in DFO-treated C2C12 cells; three genes *POLGB*, *NRF1*, and *NFE2L2* were upregulated in DFO-treated C2C12 cells; and four genes *POLGA*, *PGC1A*, *PPRC1*, and *DRP1* were unchanged in DFO-treated C2C12 cells (Figure 4B). To determine whether increased MitoBio alters iron genes, the expression of *FPN1* and *TFRC* was evaluated in the C2C12 cells overexpressed with the *PGC1A* (PGC1A-OE). The expression of *TFRC* is dramatically increased, while *FPN1* was decreased in PGC1A-OE C2C12 cells (Figure 4C). Therefore, we conclude that both cellular iron and MitoBio are positively required for cellular physiological or pathological functions.

**Figure 5:**
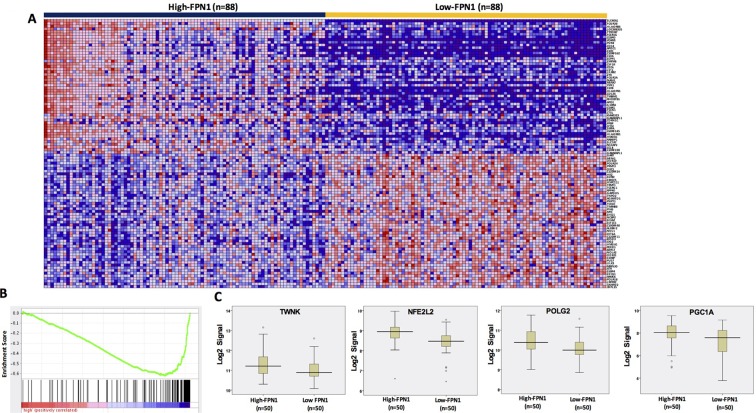
FPN1 dysregulation in MM alters MitoBio genes **(A)** A gene expression clustergram of distinct genes in plasma cells between 88 MM patients with high level or low FPN1 expression. **(B)** GSEA shows that the mitochondrial transcription pathway is significantly changed between high- and low-FPN1 expression. **(C)** Gene expression profiles of 4 MitoBio genes in 50 MM samples with high level or low of FPN1 expression (p<0.05).

**Figure 6:**
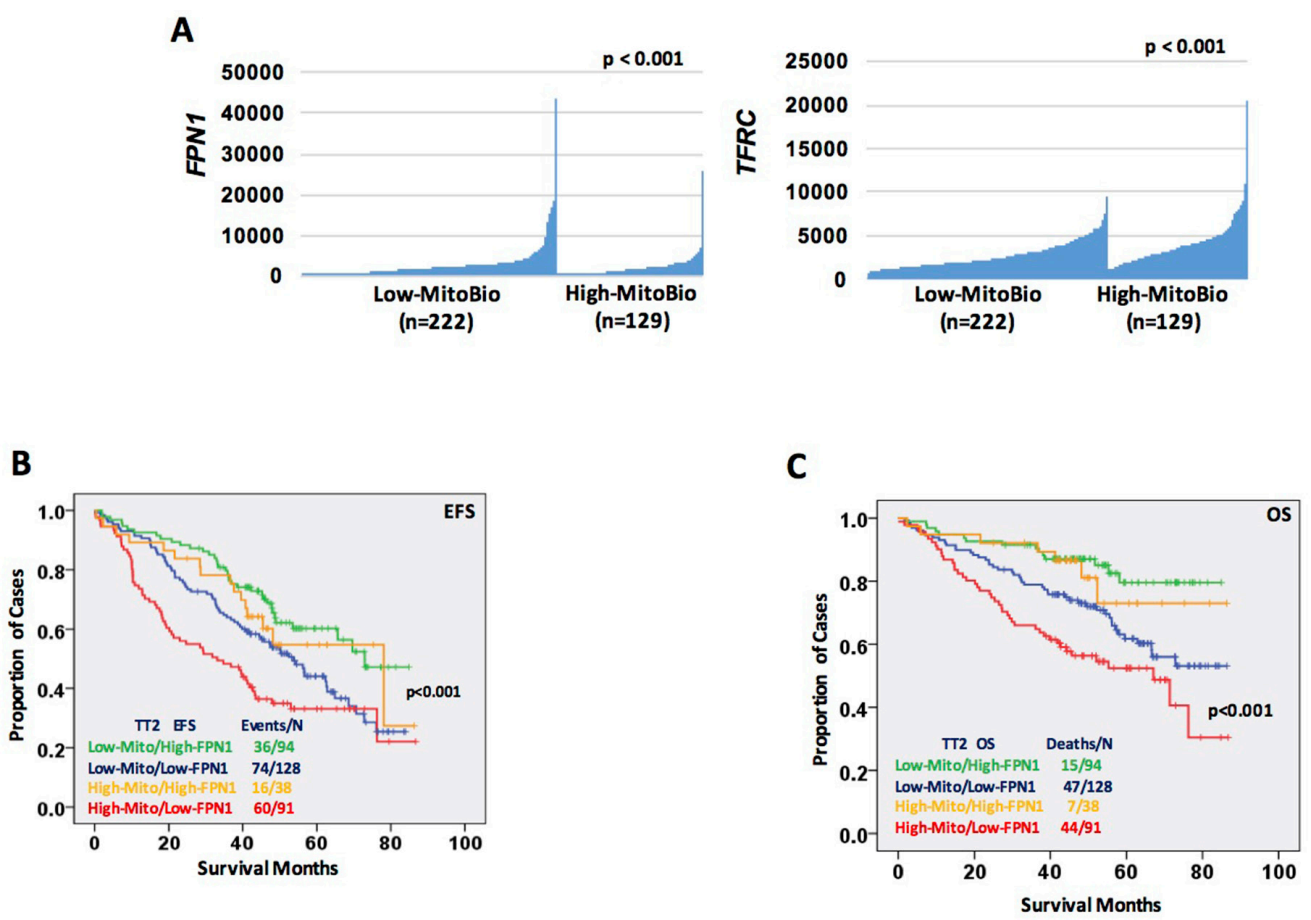
Increased cellular iron is positively correlated with dysregulated mitochondrial metabolism **(A)** Correlation of *FPN1* (SLC40A, D) and *TFRC* (E) expression within patients showing low mitochondrial-score (n=222) or high mitochondrial-score (n=129). **(B** & **C)** Kaplan–Meier analyses of free event survival (EFS, F) and overall survival (OS, G) of MM patients enrolled in TT2 cohort. Each line represents different combinations as described in the figure and color coded.

We have reported that most of iron-metabolism genes are dysregulated in MM cells [11]. Notably, *FPN1* is significantly downregulated in MM cells compared to MGUS and NPCs and linked to a poor prognosis in MM. Based on results derived from C2C12 cells, we wanted to determine whether dysregulated iron metabolism was related to an increased MitoBio in MM progression. By comparing high-FPN1 (n=88) with low-FPN1 (n=88) from 351 newly diagnosed MM samples, more than 500 genes were significantly differentially expressed between these groups and the top 100 genes are presented in the heatmap (Figure 5A). The GSEA showed the mitochondrial transcription pathway was ranked number 4 among the dysregulated pathways (Figure 5B; p < 10^-4^) between high- and low-FPN1 MM samples. Importantly, the MitoBio genes, *Twinkle*, *NFE2L2*, *POLG2*, and *PGC1A*, were negatively correlated with the expression of *FPN1* in primary MM cells (Figure 5C). We further correlate the expression of *FPN1* and *TFRC* with the mitochondrial-score in primary MM samples. As shown respectively in the Figure 6A, a high mitochondrial score was negatively correlated with the expression of *FPN1* (p < 0.001), and positively correlated with the expression of *TFRC* (p < 0.001). To determine whether increased cellular iron and increased mitochondrial score have a synergistic effect in MM progression, Kaplan–Meier analyses were performed between patients with different combinations of high- or low-FPN1 with high- or low-mitochondrial-score among the 351 newly diagnosed MM patients enrolled in the TT2 trial. The MM patients who have a high mitochondrial-score and a low-FPN1 signal show an inferior prognosis, whereas those who have a low mitochondrial-score and a high-FPN1 signal show the best prognosis in both EFS and OS (Figure 6B and C, p <0.001). Together, these data indicate that increased cellular iron is positively correlated with dysregulated mitochondrial metabolism, and synergizes for a poor outcome in MM.

### Pharmacological ascorbic acid induces myeloma cell death by targeting mitochondria

**Figure 7:**
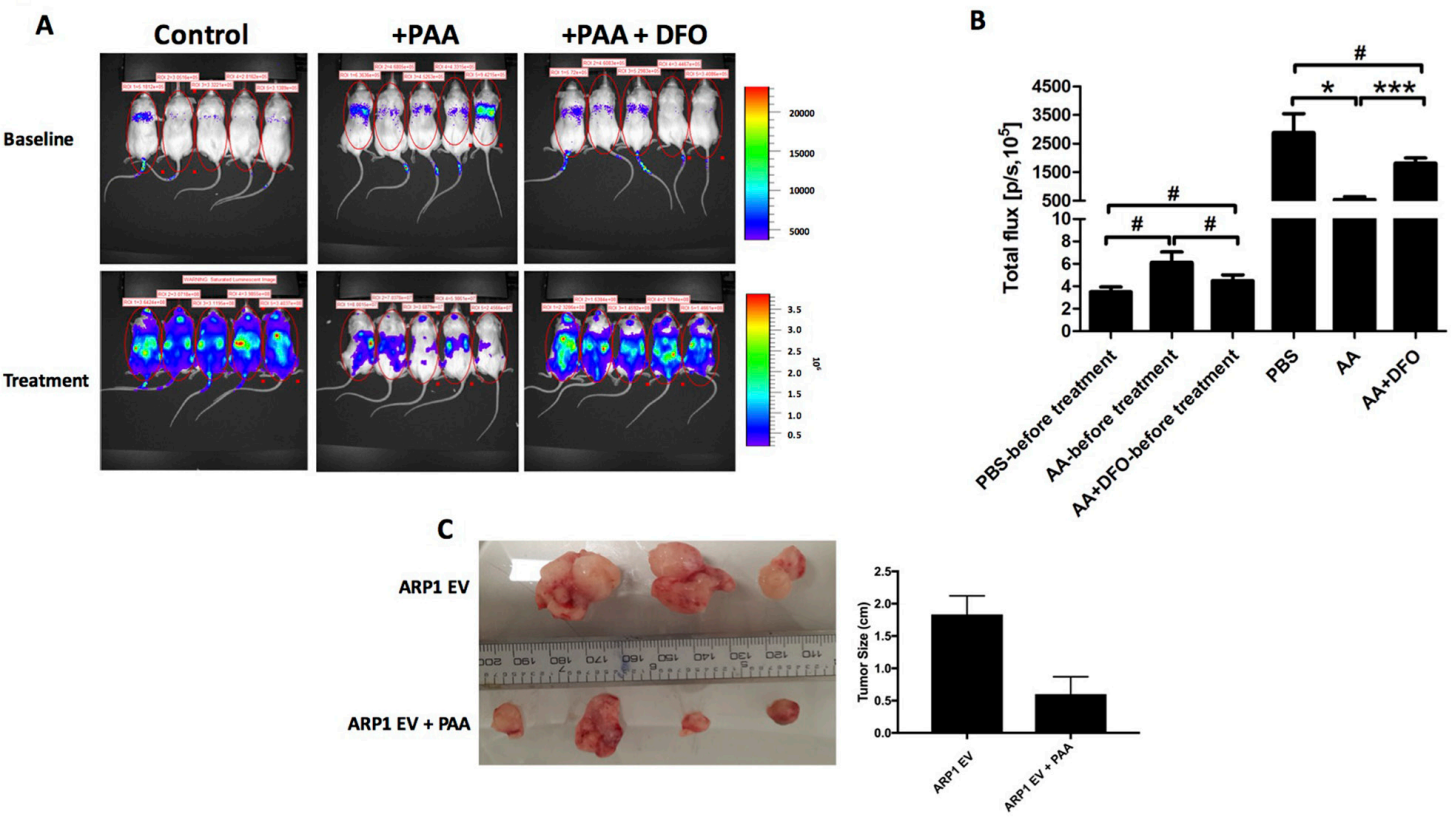
PAA prevents tumor growth in an iron-dependnet manner in MM **(A)** Xenografted NOD.Cγ -Rag1 mice (n=5/group) were treated with PAA alone and in combination with DFO. After one-week injection of ARP1 cells, mice were treated with either PAA (4mg/kg) injected intraperitoneal once a day, 5 days every week for 3 weeks. DFO (100 mg/kg) was injected intraperitoneal once a day, 2 days a week for 3 weeks. **(B)** Total flux of mice described in A indicates quantification of luciferase intensity (tumor burden) of mice pre- and post treatment at different time points. ^#^No significance, ^*^p < 0.05, ^***^ p < 0.001. **(C)** Another MM mouse model injected ARP1 intraperitoneally and treated with PAA was used and analyzed, tumors were dissected at week 3 after PAA treatment from each mouse. Tumor size was measured (right graph bar).

AIF1 is a mitochondrial FAD-dependent oxidoreductase that plays a vital role in oxidative phosphorylation and redox metabolism in normal and cancer cells [18–22]. Because the OXPHOS pathway is significantly activated in both bortezomib-resistant and relapsed MM cells, and PAA, in the presence of iron, leads to the formation of ROS resulting in AIF1 cleavage and translocation from the mitochondria to the nucleus with consequent cell death, we hypothesized that the signaling pathway of MitoBio might be involved in PAA-induced MM death. PAA, > 1mM, results in a 50- fold higher plasma levels than a physiological dose of ascorbate (1mg/kg) when administered intravenously [1]. In this study, we injected 1 x 10^6^ ARP1 MM cells intravenously in 15 NOD-Rag^null^ mice and started treating five mice with PAA 4mg/kg intraperitoneal injections (five times/week for three weeks). Five mice were treated with PAA plus the iron chelator DFO, and another five mice treated with PBS as controls. DFO (100 mg/kg) was injected intraperitoneal once a day, (two days/week for three weeks). Tumor growth was significantly inhibited in the PAA-treated mice compared to those controls (Figure 7A & B); while the combination of PAA with DFO did not show a significant difference in tumor growth compared to the control group (p> 0.05). We also repeated this study by injecting 1 x 10^6^ ARP1 MM cells subcutaneously in eight NOD-Rag^null^ mice, four mice treated with PAA, and four mice as controls. Consistent with our previous results when MM cells were injected intravenously, we observed that tumor size was significantly smaller in the PAA-treated mice compared to those controls in these xenografted mice intraperitoneally injected (one mouse died from the control group) (Figure 7C). MM tumor cells were isolated from two mice in each group and microarrays were performed.

**Figure 8:**
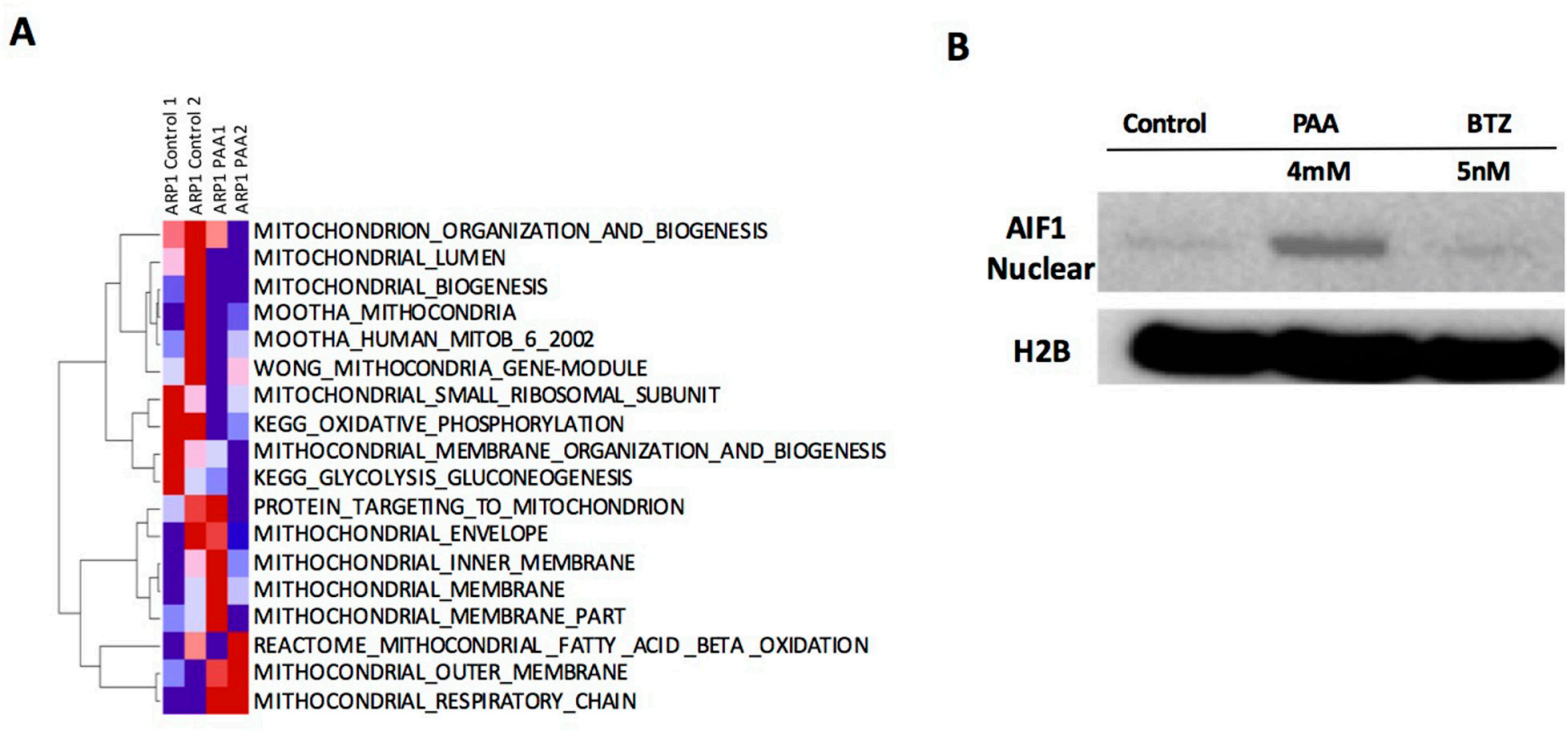
A. PAA inhibits mitochondrial oxidative phosphoryltion **(A)** Total RNA was extracted from two mice from control (control 1,2) and PAA (PAA 1,2) groups for microarray. ssGSEA analysis of 18 mitochondrial gene datasets is shown. **(B)** ARP1 cells were incubated with or without PAA (4mM) or bortezomib (BTZ, 5nM). After 1 hr PAA was washed away. After 6 hours, cellular fractionation was performed in each sample and nuclear AIF1 and H2B levels were analyzed by western blotting.

The ssGSEA analysis of 18 mitochondrial pathways indicated that PAA activates mitochondrial outer membrane and respiratory chain signaling pathways but inhibited small ribosomal subunit and oxidative phosphorylation signaling pathways (Figure 8A). We also performed western blots for AIF1 in ARP1 MM cells with or without PAA treatment and bortezomib was used as a negative control. As shown in the Figure 8B, nuclear AIF1 expression was significantly increased in PAA-treated MM cells compared to the control cells and the bortezomib-treated cells, further indicating that mitochondria were targeted by PAA. Since the oxidative phosphorylation pathway is significantly increased in both drug-resistant MM cells and relapsed MM samples, suggesting that PAA can overcome drug resistance to bortezomib by suppressing mitochondrial oxidative phosphorylation.

## DISCUSSION

Here we report that the MitoBio signature is increased in MM cells compared to MGUS and normal plasma cells. Of the 18 MitoBio genes, eight are linked to an inferior overall survival including 559 newly diagnosed MM patients. A supervised hierarchical clustering further showed that a subset of MM patients has a high MitoBio signature compared to normal and MGUS samples. In addition to analyzing the 18 MitoBio genes, we also compared mitochondrial structure and functional signaling pathways including mitochondrial lumen, small ribosomal subunit, targeting to mitochondrion, organization and biogenesis, envelop, inner membrane, outer membrane, respiratory chain, oxidative phosphorylation, fatty acid ß-oxidation, and glycolysis gluconeogenesis between normal plasma cells and MM cells. As expected, the MM patients who have an increased MitoBio-related score show an early disease relapse and premature death. This is consistent with the report that melanoma patients with higher expression of MitoBio and tumor metabolism had worse overall survival [23]. Interestingly, all mitochondrial structure signaling pathways, and MitoBio and oxidative phosphorylation signatures are significantly upregulated in MM cells compared to normal plasma cells (p< 0.001); while the signaling pathways of fatty acid ß-oxidation and glycolysis gluconeogenesis do not have a difference between normal and tumor plasma cells. The supervised cluster in Figure 2b shows clearly that both MitoBio and oxidative phosphorylation signatures are classified together with the mitochondrial structure signatures, but distinguished from fatty acid ß-oxidation and glycolysis gluconeogenesis pathways. Although both oxidative phosphorylation and aerobic glycolysis play an important role in generating ATP for tumor cell growth, our data suggest that oxidative phosphorylation is the main pathway to provide energy for MM cell growth and proliferation.

To determine whether mitochondrial biogenesis is associated with disease relapse, ssGSEA analyses were performed on 51 paired MM samples collected at diagnosis and at relapse. Seven of 18 pathways were significantly upregulated in relapsed MM samples compared to those at diagnosis. To further determine whether this MitoBio is also related to drug resistance in 169 relapsed MM samples treated by bortezomib, eight of 18 pathways were increased in bortezomib-resistant MM samples, and five of them are commonly increased in both drug resistant and relapsed MM samples. We found that the direct MitoBio signature is only increased in bortezomib-resistant MM samples and not in the relapsed MM samples. However, the oxidative phosphorylation pathway is increased in both relapsed and drug resistant MM cells, supporting that the ATP provided by oxidative phosphorylation is not only important for MM disease development but also important for drug resistance and disease relapse in MM. We recently reported that PAA leads to the formation of ROS, resulting in AIF1 cleavage and translocation from the mitochondria to the nucleus, causing cell death. It is known that PAA induces cancer cell death and is dependent on depletion of ATP [24]. We compared these 18 mitochondrial signaling pathways before and after PAA treatment in MM cell line ARP1. We found that the OXPHOS signaling is one of the four pathways inhibited. Our data suggest that PAA inhibits OXPHOS signaling pathway and may cause a depletion of ATP resulting in MM cell death. PAA will be important to target drug resistant and relapsed MM in clinic.

The transcription co-activators, PGC1A and PGC1B, are the major proteins that enhance mitochondrial biogenesis, oxidative phosphorylation and the oxygen consumption rate [25, 26]. c-Myc induces MitoBio through the activation of many MitoBio genes, especially dependent on PGC1B. PGC1A positively regulates a subset of OXPHOS genes in a time-dependent manner in a mouse skeletal muscle cell line [25]. Iron is essential for MitoBio and the iron importer gene transferrin receptor (*TFRC*) is a direct target of c-Myc. In our study, we found a positive correlation between cellular iron and the MitoBio signature. Depletion of cellular iron by DFO in C2C12 cells caused the expression of most MitoBio genes to decrease, while overexpression of PGC1A in C2C12 cells downregulated the expression of iron exporter gene *FPN1* and upregulated the expression of TFRC. Consistently, MM samples with low FPN1 expression increased the expression of *TWNK*, *NFE2L2*, *POLG1*, and *PGC1A*, activated the mitochondrial transcription pathway. Importantly, MM patients with high MitoBio and low FPN1 had the worst clinical outcomes, indicating that iron is essential for MitoBio in MM cells for their biological functions, such as cell growth, proliferation and drug resistance. The possible reasons may be that both functional mitochondria and iron-sulfur cluster maintain nuclear genome stability [27], which are controlled by c-Myc in MM cells. We have recently shown that c-Myc also regulates the chromosomal instability (CIN) and drug resistant gene NEK2 in MM [28–30]. Our future work will explore whether c-Myc plays the key role in the dysregulated CIN in MM, MitoBio and iron metabolism, as well as how these pathways interact with each other.

Recent studies indicate that tumor cell growth and migration are fueled by enhanced mitochondrial biogenesis. Therefore, targeting MitoBio could be a strategy in cancer therapy. Our previous study showed that high-dosed ascorbic acid selectively kills MM cells including both primary MM cells and MM cell lines. In this study, we further determined that PAA inhibited MM cell growth dependent on cellular iron using an *in vivo* xenografted MM mouse model. This response process by PAA administration leads to mitochondrial swelling, the cristae disappeared and AIF1 cleavage and nuclear translocation was demonstrated by transmission electron microscopy (TEM) [1]. AIF1 is a mitochondrial FAD-dependent oxidoreductase, that plays a vital role in oxidative phosphorylation and redox metabolism in normal and cancer cells [18–22]. It is very likely that PAA generates high cellular ROS and inhibits mitochondrial AIF1 function as an oxidoreductase resulting in inhibition of oxidative phosphorylation and ATP depletion. In addition, AIF1 is an intermembrane space (IMS) component of the mitochondrial and characterized as a pro-apoptotic gene [31, 32]. The nuclear AIF1 or truncated AIF1 induced by PAA initiates chromatolysis and apoptosis-independent cell death [33]. Our data show that the proteasome inhibitor bortezomib, a widely-used drug in MM, has no effect on AIF1 expression, cleavage and localization; suggesting that PAA could be applied in MM patients, who are resistant to bortezomib. Targeting mitochondria proteins, such as CDDO and 10-TPP, etc., have been shown to enhance the chemotherapeutic effects in pre-clinical and clinical MM studies [2, 34, 35]. Future work will focus on the selection of those promising agents which have specific toxicity to MM cells but spare normal tissues.

## MATERIALS AND METHODS

All methods were carried out in accordance with relevant institutional guidelines and regulations at the University of Iowa.

### Patient samples

Gene expression profiles (GEP) of highly purified bone marrow plasma cells were analyzed from 559 newly diagnosed patients with MM including 351 from total therapy 2 (TT2) and 208 from TT3 cohorts. All consented patients’ samples were collected according institutional approval. The protocol was approved by the institutional review board and the Food and Drug Administration as earlier described [36, 37]. The Gene Expression Omnibus database accession numbers described in this article were retrieved from GSE2658, GSE5900 and GSE9782. Kaplan–Meier analyses of event-free survival (EFS) and overall survival (OS) about patients in the TT2 and TT3 cohorts were performed using methods implemented in the SurvExpress website. Univariate analysis was used to test the associations between those mitochondrial biogenesis biomarkers and 19 clinical parameters in newly diagnosed myeloma patients.

### Gene set enrichment analysis (GSEA) and ssGSEA

We performed GSEA and ssGSEA by using the applications available at the GenePattern platform of the Broad Institute of MIT and Harvard (https://genepattern.broadinstitute.org/gp/pages/index.jsf). For GESA, we estimated the signal-to-noise ratio and false discovery rates (FDR), from 1000 gene-set permutations. We tested the Gene ontology: Biological Process terms and KEGG pathways gene sets. For ssGSEA analysis, gene set activation scores for each sample were generated by online ssGSEA module based on the methodology described in Barbie et al., 2009 [38]. Raw enrichment scores were subjected to Hierarchiacal-Clustering analysis and heatmaps were generated by Hierachical-Clustering-Image module from Genepattern platform.

### Gene expression profiling (GEP)

mRNA was extracted from ARP1 cell line before and after high-dosed ascorbic acid treatment using RNeasy kit (Qiagen, Valencia, CA, USA) accordingly with the manufacturer’s instruction. GEP was performed on the Affymetrix Plus2 chips on the 7G workstation and data was analyzed as previously reported [13, 39].

### Human myeloma cells in NOD.Cγ-Rag1 mice

The animal study was performed according to the guidelines of the Institutional Animal Care and local veterinary office and ethics committee of the University of Iowa, USA under approved protocol (IACUC 5081482). NOD.Cγ-Rag1 mice 6–8 weeks old (Jackson Laboratory, Bar Harbor, ME, USA) were injected intravenously or intraperitoneally injection with ARP1 MM cells (1 × 10^6^) expressing luciferase. One-week after injection of ARP1 cells, mice were treated with either PAA (4 mg/kg) intraperitoneally injection once a day, 5 days every week for 3 weeks. DFO (100 mg/kg) was injected intraperitoneally once a day, 2 days a week for 3 weeks. The mice were euthanized when a humane endpoint was reached.

### Cell culture

Human myeloma ARP1 cell line (kindly provided by Dr. John Shaughnessy, University of Arkansas for Medical Services, AR) was cultured in RPMI 1640 (Invitrogen, Carlsbad, CA, USA), supplemented with 10% heat-inactivated FBS (Invitrogen), penicillin (100 IU/mL), and streptomycin (100 μg/mL) in a humidified incubator at 37 °C and 5% CO_2_/95% air.

### Western blotting

Cells were harvested and lysed with lysis buffer: 150 mM NaCl, 10 mM EDTA, 10 mM Tris, pH 7.4, 1% X-100 Triton. Cellular fractionation was performed according manufacturer’s instruction (FractionPREP, BioVision). Cell lysates were subjected to SDS-PAGE, transferred onto a pure nitrocellulose membrane (BioRad) and blocked with 5% fat-free milk. Primary antibodies for immunoblotting included: anti-AIF1 (1:1000, Cell Signaling) and and anti-H2B (1:1000, Cell Signaling) as loading control. Membranes were incubated with horseradish peroxidase (HRP)-conjugated anti-mouse secondary antibody (1:10,000, Santa Cruz Biotechnology, cat#: sc-2005) or anti-rabbit secondary antibody (1: 10,000, AnaSpec Inc., cat#: AS-28177) for 1 h and chemi-luminescence signals were detected by HRP substrate.

## Abbreviations

MM: Multiple Myeloma
PAA: Pharmacological Ascorbic Acid
AIF1: Apoptosis-Inducing Factor 1
OXPHOS: oxidative phosphorylation
GEP: Gene Expression Profiles
MitoBio: Mitochondrial biogenesis
MGUS: Monoclonal gammopathy of undetermined significance
NPC: Normal Plasma Cells
